# An Unusual Case of Hemolytic Disease of Newborn Due to ABO and Rh Isoimmunization

**DOI:** 10.7759/cureus.12121

**Published:** 2020-12-17

**Authors:** Suman S Routray, Jagdish P Sahoo, Rachita Behera, Devi Acharya, Girija N Kanungo

**Affiliations:** 1 Transfusion Medicine, All India Institute of Medical Sciences, Bhubaneshwar, Bhubaneswar, IND; 2 Neonatology, Institute of Medical Sciences (IMS) and SUM Hospital, Bhubaneswar, IND; 3 Transfusion Medicine, Institute of Medical Sciences (IMS) and SUM Hospital, Bhubaneswar, IND; 4 Transfusion Medicine, AMRI Hospital, Bhubaneswar, IND

**Keywords:** hemolytic disease of the newborn, abo incompatibility, rh isoimmunisation, hyperbilirubinemia

## Abstract

Anti-D is the most common cause of hemolytic disease of the newborn (HDN) in the developing countries even after the introduction of anti-D immunoprophylaxis. Still, ABO incompatibility and other alloantibodies against minor blood group antigens have emerged as significant causes of HDN. Moreover, ABO incompatibility acts as a protective barrier to the expression of Rh isoimmunization. Here we are presenting a case of HDN where both Rh and ABO incompatibility co-existed with their manifestations in a B positive neonate born to an O positive mother. Use of appropriate elution technique can aid in the diagnosis of such cases. Hence, antenatal screening of all mothers irrespective of their Rh D status can help in early diagnosis and proper management that can decrease the neonatal morbidity and mortality.

## Introduction

Hemolytic disease of the newborn (HDN) occurs as a result of immune-mediated destruction of red cells leading to increase mortality and morbidity in neonates. ABO incompatibility has emerged as a significant cause of HDN in the western world after the introduction of prophylactic anti-D administration [1]. Studies have demonstrated that in the presence of major ABO incompatibility between mother and fetus, the incidence of D isoimmunization is less in mother. This is apparently due to the hemolysis of ABO-incompatible red cells of the fetus in the mother’s circulation before the recognition of the Rh incompatible antigen by the mother’s immune system. Here we report a case of a hemolytic disease of newborn caused by the coexistence of both ABO and Rh incompatibility.

## Case presentation

A multigravida mother (G5A4L0) with the bad obstetric history presented at 32nd week of gestation to our hospital with the complaint of decreased fetal movement. She had a history of receiving 11 transfusions during her past abortions. Because of fetal distress, emergency caesarian section (LSCS) was performed, and a baby girl weighing 1.9 kg was delivered. The baby was transferred to the neonatal intensive care unit (NICU) on day 1 for preterm care and was started on full feeding. She developed anaemia and jaundice on day 3 during her stay at NICU.

On routine investigations, the laboratory findings were as follows: total serum bilirubin was 11.3 mg/dl, Hb 14.3 gm/dl and reticulocyte count 4.8%. Peripheral smear reported features of hemolytic anaemia. G6PD estimation was performed and found to be 14.8 units/g of Hb (within standard limit). Thyroid profile was normal. Because of suspected HDN, a blood sample was sent to the department of transfusion medicine for immunohaematological work up.

**Figure 1:**
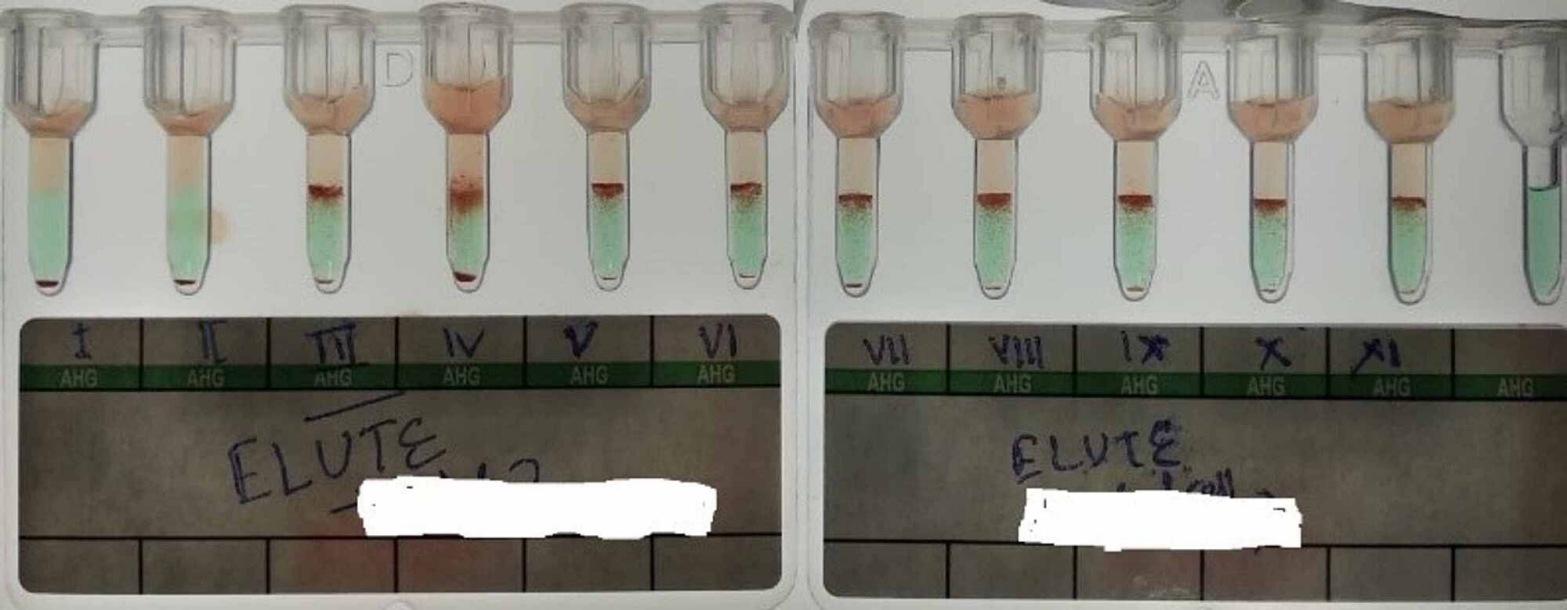
Antibody Identification of Neonate’s Eluate Showing Anti-c Specificity.

The forward blood group of the neonate was B Rh D positive with the gold standard tube technique, and that of the mother was O Rh D positive by both forward and reverse grouping. Polyspecific direct antiglobulin test (DAT) by column agglutination technique (Anti Human Globulin gel card containing IgG + C3d component, BIORAD) showed 4 + reaction. Monospecific DAT using IgG + C3d gel card (Tulip Diagnostics) showed IgG specificity with C3d and control being negative. Glycine-hydrochloride acid elution was done using Gamma Elu-Kit (Biorad, Switzerland). The eluate from DAT positive red cells was tested against commercialized available 0.8% three cell panel (DIACELL I-II-III, Biorad, Switzerland). It was found to be reactive in panel II and III. On antibody identification using commercially available 11 cell panel (Biorad, Switzerland), anti-c was detected (Figure 1, Table 1).

**Table 1:** Antibody Identification Panel for Eluate of Neonate Showing Anti-c Specificity.

	Rh-hr						Kell						Duffy		Kidd		Lewis		P1	MNS				Luth		Xga	AHG
	D	C	E	c	e	Cw	K	k	Kpa	Kpb	Jsa	Jsb	Fya	Fyb	JKa	JKb	Lea	Leb	P1	M	N	S	s	Lua	Lub		
1 CCC^w^D.ee R_1_^w^R_1_	+	+	0	0	+	+	0	+	0	+	nt	nt	+	0	0	+	0	0	+	+	+	+	+	0	+	+	0
2 CCD.ee R_1_R_1_	+	+	0	0	+	0	+	+	0	+	nt	nt	0	+	+	0	+	0	0	0	+	0	+	0	+	+	0
3 ccD.EE R_2_R_2_	+	0	+	+	0	0	0	+	0	+	nt	nt	+	+	+	0	0	+	+	+	0	+	0	0	+	+	4+
4 Ccddee r’r	0	+	0	+	+	0	0	+	0	+	nt	nt	+	0	+	+	0	+	0	+	0	+	0	0	+	+	4+
5 ccddEe r”r	0	0	+	+	+	0	0	+	0	+	nt	nt	+	+	0	+	+	0	0	0	+	+	+	0	+	+	4+
6 ccddee rr	0	0	0	+	+	0	+	+	0	+	nt	nt	0	+	0	+	0	+	+	+	0	+	+	+	+	+	4+
7 ccddee rr	0	0	0	+	+	0	0	+	0	+	nt	nt	+	0	+	0	0	+	0	0	+	0	+	0	+	+	4+
8 ccD.ee R_0_r	+	0	0	+	+	0	0	+	0	+	+	nt	0	0	+	+	+	0	+	+	0	+	+	0	+	0	4+
9 ccddee rr	0	0	0	+	+	0	0	+	+	+	nt	nt	+	+	+	0	+	0	+	+	0	+	+	0	+	nt	4+
10 ccddee rr	0	0	0	+	+	0	0	+	0	+	nt	nt	0	+	+	0	0	+	+	+	+	0	+	+	+	nt	4+
11 ccddee rr	0	0	0	+	+	0	0	+	0	+	nt	nt	0	+	0	+	0	0	0	+	0	+	0	0	+	nt	4+

**Figure 2:**
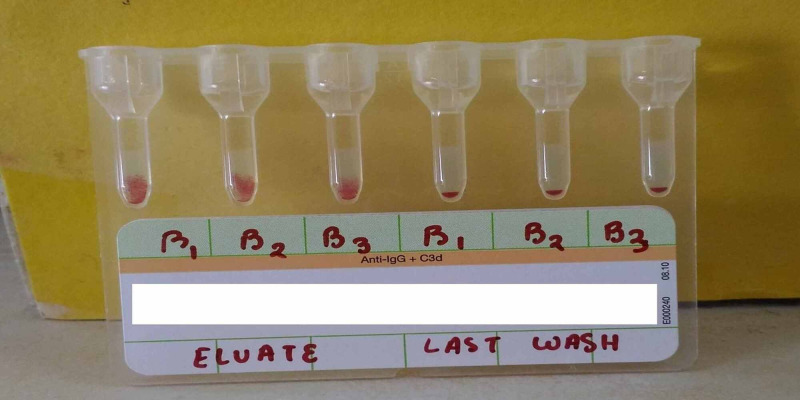
Antibody Identification of Neonate’s Eluate Showing Anti-B Specificity.

Presence of anti-E was ruled out by using select cells (c-E+, c+E-). Heat elution at 560 C for 10 minutes using 6% albumin with intermittent agitation on the neonate red blood cells was performed. The eluate, when tested against freshly prepared 5% cell suspension of c negative A, B and O pooled cells, showed specificity to anti-B at AHG phase confirming the diagnosis of ABO HDN (Figure 2).

To confirm the anti-c alloantibody, which was transferred from the mother, maternal serum was tested for antibody screening and identification. Anti-c was detected with a titre of 16 at AHG phase. Anti-B titre was 1048 at AHG phase.

On extended RH-Kell phenotyping, the mother was found to be C+ c- E- e+, K-, father C- c + E- e +, K - and that of the baby was C- c+E- e+, K-. Baby received phototherapy and was discharged with a stable vitals.

## Discussion

Here we have reported a rare case of HDN where hemolysis of red cells was due to anti-c and anti-B. Many works of the literature suggest that ABO incompatibility protects somewhat against Rh isoimmunization. The maternal IgG antibodies that cross the placenta causing HDN could be naturally occurring (Anti A, Anti B) or immune antibodies developed following sensitization event. This happens in 15-20% of all pregnancies. In the case of ABO HDN, the fetal red cells were destroyed by the maternal reticuloendothelial system as soon as the fetal red cells entered the maternal circulation by the natural occurring anti-A/anti-B. So the development of an antibody against Rh antigen is not possible. A high titer of these immune antibodies may not present with adverse effects in utero as A and B antigens are present on cells of all other tissues and body fluid and not only on red cells. The presence of these antigens helps to protect the incompatible fetal red cells by neutralizing the transferred maternal antibody with small amounts of an antibody reacting directly with the fetal red cells [2]. The red cells which are sensitized by the antibodies are destroyed by macrophages in the fetal spleen with consequent hyper-bilirubinemia in about 10% of cases [3,4]. With the widespread use of RhD immunoglobulin, the focus has shifted to the non-RhD antibodies causing isoimmunization. Other Rh antigens include C, c, E, and e antigens. DCe is the most common haplotype in Caucasians (42%), Native Americans (44%), Asians (70%) [5], and also among Indians (40.9%) [6].

The severity of hyperbilirubinemia (total bilirubin 14.3 gm/dl) is more in Rh HDN in comparison to ABO HDN [7]. In this case, the previous history of multiple transfusion and abortion was a concern for alloantibodies (Rh/Non-Rh HDN). So glycine-acid elution was performed and from the elute anti-c was detected. Whether the development of Rh alloimmunization (anti-c) in mother has occurred as a result of previous pregnancies or transfusions was inconclusive. Around 3.77% of pregnant mothers with bad obstetric history have a risk of developing alloimmunization [8]. Red cell alloimmunization among expectant mothers is widely studied around different parts of the world, ranging from 0.4% to 2.7% and anti-E, anti-c, anti-kell are the most common alloantibodies among Rh D positive pregnant women [9-13]. Anti-c was identified to cause severe HDN in about 10% of pregnancies with c+ foetus in Netherland [11]. Anti-E, anti-C and anti-e are generally implicated with a mild form of Rh HDN [14]. Anti-c is the second most antibody commonly encountered in Rh D positive Indian mothers with high-risk pregnancies or with a history of transfusion that affects fetus resulting in HDN [15]. ABO HDN is more common among neonates of O blood group mothers as naturally occurring IgG anti-A/anti-B is more prevalent with a significant high titre [16]. In our case, the mother was O positive, and the baby was B positive. So ABO HDN was in suspicion. So heat elution was performed, and anti-B was detected in elute. Both heat elution and glycine acid elution confirmed the case to be the existence of both ABO and Rh HDN. Some literature also mentioned about the utility of heat elution in the detection of Rh/Non-Rh alloantibodies though not superior to glycine-acid elution [17]. Presence of c antigen on RBC of father/baby and absence on RBC of the mother was also a corroborative finding to support the development of anti-c in the mother which transferred to the baby through the placenta.

Mina et al. reported a case of HDN due to Rh anti-c in an infant of an Rh-positive mother, who required double volume exchange transfusion (DVET) [18]. Here the patient was managed by phototherapy only without the need of DVET. This could be explained by the fact that ABO HDN might have masked the effect of Rh alloimmunization.

## Conclusions

The diagnosis, acute management, and follow‐up of neonates with HDN still represent an important area of activity for maternity/neonatal services. With the introduction of RH D immunization, the chances of Rh D HDFN has been on reversal trend. Consequently, hemolytic disease due to ABO incompatibility and other alloantibodies along with Rh alloantibodies have taken centre stage. Antibody screening regularly of all pregnant women is now implemented in many developed countries as a part of the national program, which aids in the appropriate management. All the pregnancies irrespective of Rh D status should be screened in developing countries like India to reduce the morbidity and mortality related to HDN. Moreover, early detection of antibody aids in the appropriate transfusion support with the antigen-negative unit if needed.
